# Bio-dissociable platinum quantum dot cluster nanozymes for nonhormonal endometriosis therapy through microenvironment remodeling

**DOI:** 10.1016/j.mtbio.2026.103785

**Published:** 2026-10-26

**Authors:** Cheng Cao, Jie Ding, Huifang Hao, Yingao Jiao, Yu Cao, Yanfei Fu, Xinyuan Cui, Dengyao Gao, Yan Gao, You Wang, Chaoqin Yu, Ting Wang, Yanlei Liu

**Affiliations:** a Institute of Intelligent Health Diagnosis and Treatment, School of Automation and Intelligent Sensing, Shanghai Jiao Tong University, Shanghai, 200240, People's Republic of China; b Department of Traditional Chinese Medicine, The First Affiliated Hospital of Naval Medical University, Shanghai, 200433, People's Republic of China; c Obstetrics & Gynecology Hospital of Fudan University, Shanghai Key Lab of Reproduction and Development, Shanghai Key Lab of Female Reproductive Endocrine Related Diseases CN, Shanghai, 200011, People's Republic of China; d Shanghai Key Laboratory of Gynecologic Oncology, Renji Hospital, School of Medicine, Shanghai Jiao Tong University, Shanghai, 200127, People's Republic of China

**Keywords:** Endometriosis, Nanozymes, Inflammation, Pt quantum dots, Nonhormonal therapy

## Abstract

Endometriosis (EMS) is a chronic inflammatory disorder marked by ectopic endometrial lesions that commonly cause pain and infertility. Existing surgical and hormonal therapies are limited by adverse effects and high recurrence rates, while nanozyme-based approaches offer opportunities to develop alternative therapeutic strategies. Here, we developed bio-dissociable platinum quantum dot clusters (PtQDC) as nanozymes for nonhormonal EMS therapy. PtQDC undergoes gradual dissociation in lesion tissue fluid and exhibits sustained scavenging activity toward reactive oxygen and nitrogen species. The resulting shift of endometriotic cells from their highly oxidative state beyond a favorable redox window was accompanied by impaired energy metabolism, mitochondrial dysfunction, and apoptosis. In the mouse model of EMS, PtQDC reduced lesion number and volume, remodeled the peritoneal microenvironment, inhibited pathological angiogenesis, and attenuated pain-associated signaling. PtQDC also reduced proinflammatory factors, increased IL-10, and modulated macrophage phenotypic profiles. RNA-seq analysis further revealed downregulation of Ptgs2, Mmp3, and Postn, genes associated with inflammation, invasion, and fibrosis. Thus, PtQDC integrates antioxidant, anti-inflammatory, antifibrotic, and antiangiogenic activities as a potential multitarget nonhormonal therapy for EMS.

## Graphical abstract

**Figure:**
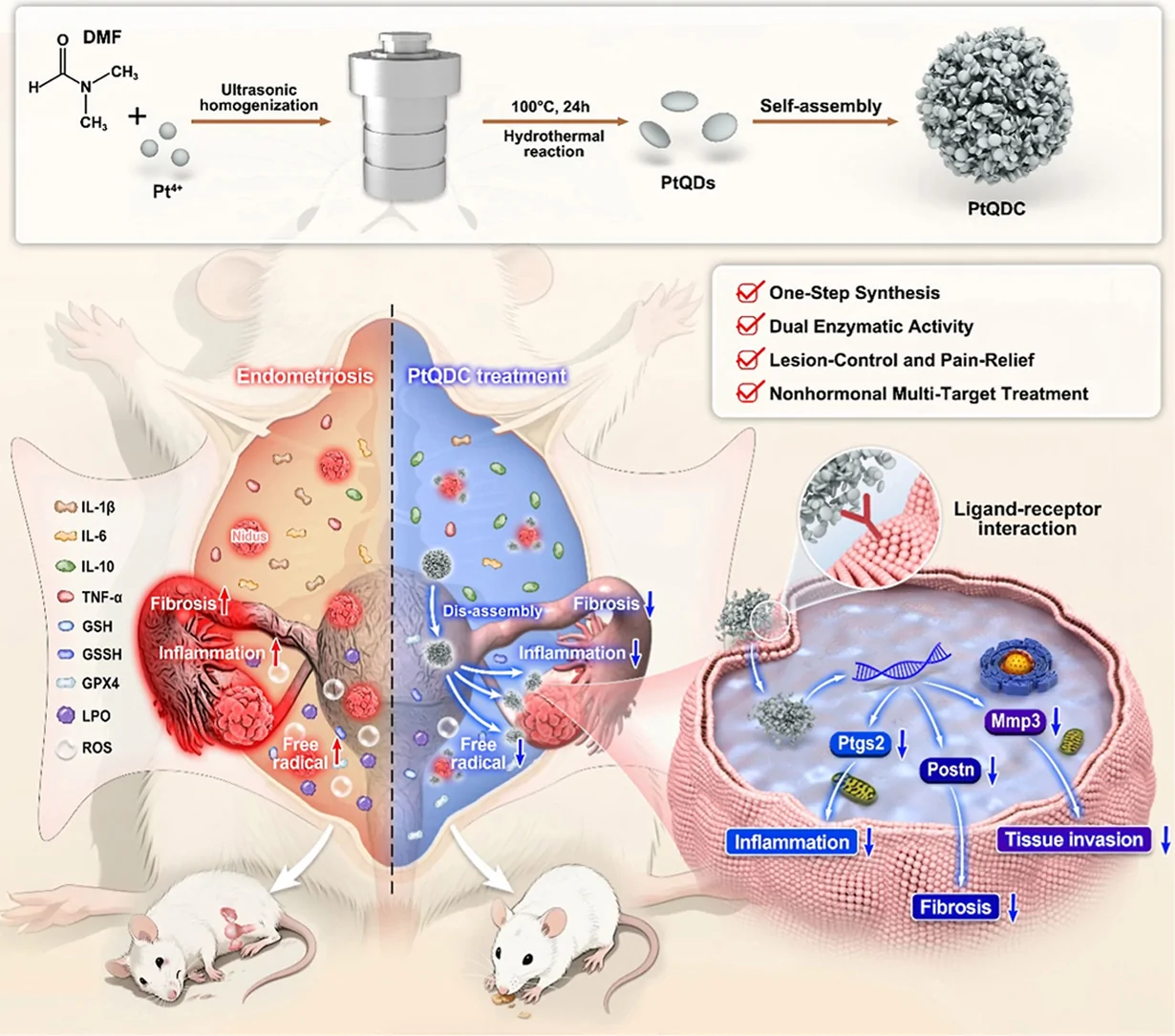

## 1 Introduction

Endometriosis (EMS) is an estrogen-dependent, benign, tumor-like chronic inflammatory gynecological disease characterized by the emergence, growth, and infiltration of both glandular and mesenchymal endometrioid tissue outside the uterine cavity. EMS is common and affects approximately 10% of women of reproductive age worldwide, with the total number of affected individuals estimated to be 190 million [1]. The prevalence of comorbid EMS in women with infertility can reach 20% - 50%, and among patients with chronic pelvic pain, the prevalence reaches 71% - 87%.

The pathogenesis of EMS is complex and has not been fully elucidated, but it is currently thought to be related to genetic, hormonal, immunologic, and inflammatory factors [2]. The mainstream theories include the retrograde menstruation theory, the body cavity epithelial chemotaxis theory, the vascular and lymphatic metastasis theory, and the stem cell theory. Recent research has suggested that genetic and epigenetic alterations, estrogen-dependent inflammation, progesterone resistance, an imbalance between cell proliferation and apoptosis, angiogenesis, neurogenesis, and tissue remodeling play important roles in the development of this disease [3]. Among them, the inflammatory response is considered among the central components, and the growth of ectopic lesions is closely associated with intraabdominal inflammation, immune alterations, and recurrent hemorrhage, which ultimately lead to fibrosis [4], [5]. Oxidative stress is also thought to be a key mechanism driving the onset of EMS and leading to progesterone resistance [6].

Owing to its unknown etiology and lack of curative treatments, the clinical management of EMS is challenging and delays in diagnosis are common, with an average of 5 to 12 years from symptom onset to diagnosis [1]. Current therapeutic goals are primarily lesion reduction, pain relief, improvement in reproductive function and prevention of recurrence. However, existing treatments have many limitations; for example, drug therapy may be accompanied by adverse events and high recurrence rates after drug discontinuation, while surgical treatment can eliminate lesions but may damage ovarian function, and the risk of recurrence after surgery remains [7], [8]. Therefore, exploring new, more effective treatment strategies with fewer side effects, such as immunotherapy, targeted therapies, and nanotechnology-based therapies, has become a hot topic of current research [4], [9], [10].

Nanozymes, as a class of nanomaterials with natural enzyme-like catalytic activity [11], [12], [13], have shown broad prospects in the biomedical field because of their excellent stability, tunable activity and easy functionalization [14], [15], [16], [17], [18], [19], [20]. Among them, platinum-based nanozymes are outstanding at efficiently scavenging oxidative radicals such as reactive oxygen species (ROS) and reactive nitrogen species (RNS) and mitigating oxidative stress because of their excellent catalase (CAT)- and superoxide dismutase (SOD)-mimicking activities [21], [22], [23], [24], [25], [26]. Nanotechnology-based approaches to EMS have explored nanozyme-mediated estradiol scavenging [27], fluorescence imaging-guided photothermal ablation [28], and surgical navigation [29], motivating further exploration of strategies targeting the oxidative and inflammatory microenvironment. Given that the core pathological features of EMS include estrogen-dependent chronic inflammation, recurrent bleeding-induced oxidative stress bursts, and the resulting aberrant neuroangiogenesis and tissue fibrosis, oxidative stress is considered a key hub connecting upstream triggers with downstream pathological effects [30], [31]. The powerful free radical scavenging ability of platinum nanozymes, on the other hand, may provide a possible pathway to intervene in this complex pathological cascade by scavenging excessive ROS in the local microenvironment and attenuating the oxidative stress-mediated sustained activation of the inflammatory pathway [32]. These effects, in turn, may inhibit the release and activity of profibrotic and proangiogenic factors, thus potentially hampering fibrosis and the construction of abnormal vascular networks. However, although the potential and advantages of platinum nanozymes for the treatment of certain chronic inflammatory diseases have been recognized, the feasibility and efficacy of their application in the treatment of EMS, that is, their sequential mechanisms of action from scavenging ROS to inhibiting inflammation and ultimately the inhibition of fibrosis and angiogenesis, remain underexplored.

It was therefore proposed to construct a Pt quantum dot cluster nanozyme, PtQDC, to produce anti-inflammatory effects and modulate the environment of endometriotic lesions for nonhormone-dependent treatment of this disease, with a bio-dissociable cluster architecture and broad reactive species-scavenging activity (Scheme 1). In vitro and in vivo experimental models were used to validate the ability of PtQDC to scavenge toxic oxidative free radicals, inhibit the release of proinflammatory factors and promote the expression of anti-inflammatory factors while modulating the immune activity of macrophages. In addition, bioinformatics was used to analyze how PtQDC regulates the microenvironment of endometriotic lesions and to observe its effects on pathological processes such as inflammation, neovascularization, and endothelial fibrosis. The effect of PtQDC on pathological inflammation caused by strong oxidative stress in endometriotic lesions, which promotes the progression of neovascularization and endothelial fibrosis and leads to lesion formation, was confirmed. This study provides a new therapeutic strategy with clinical translational potential for nonhormonal EMS treatment.

**Scheme 1:**
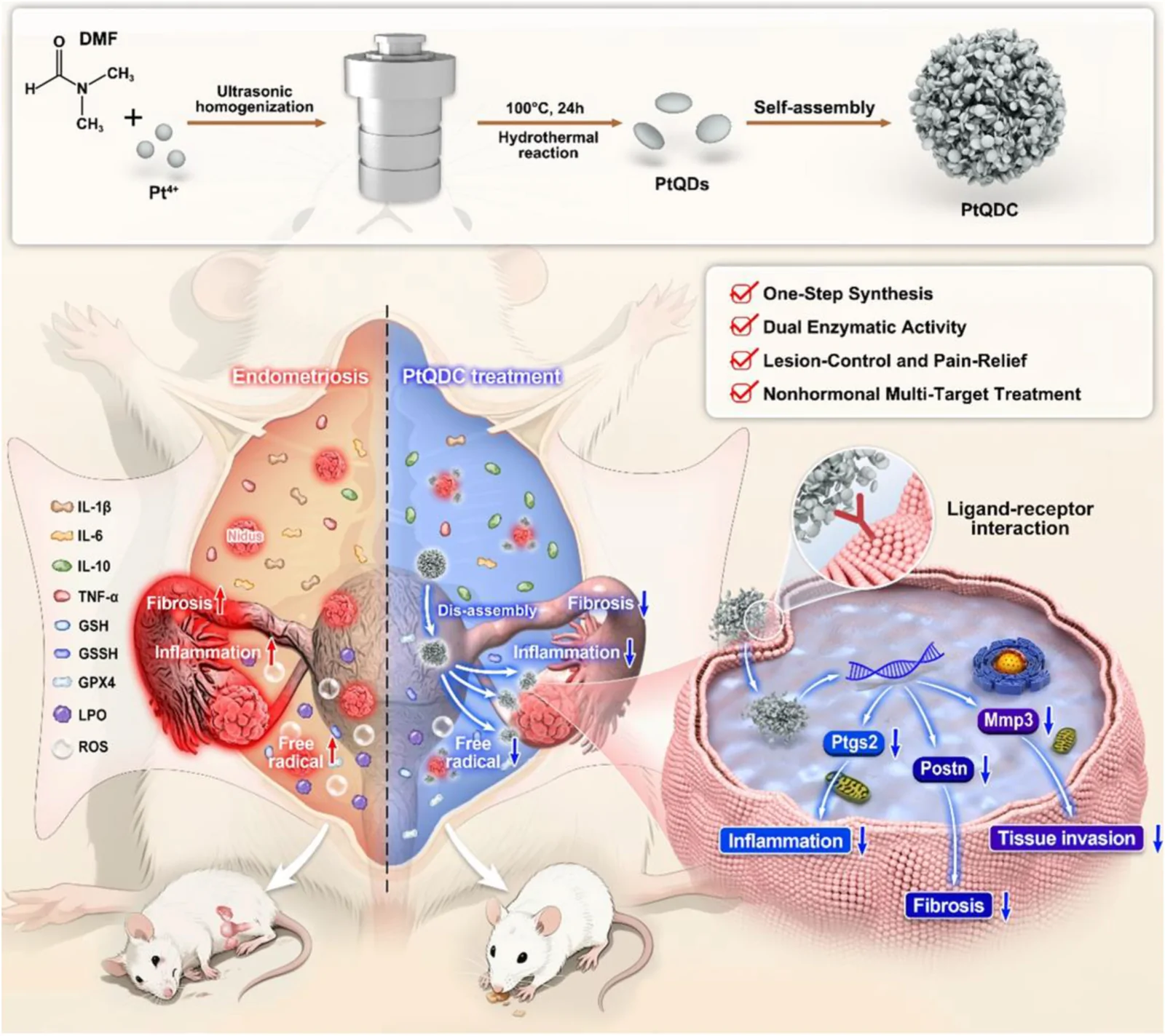
Schematic illustration of PtQDC-mediated multitarget nonhormonal therapy for endometriosis.

## 2 Results and discussion

### 2.1 Characterization of PtQDC

PtQDCs were synthesized via a one-step hydrothermal method. TEM results in Fig. 1A revealed PtQDC nanoclusters with diameters of 50–100 nm, while higher-magnification images resolved the constituent Pt quantum dots. Characterization of the lattice spacing through high-resolution TEM revealed a lattice spacing of 0.22 nm, as shown in Fig. 1B, which corresponds to the Pt(1 1 1) crystal plane. The UV‒Vis spectrum (Fig. S1) demonstrated that PtQDC exhibits broad-spectrum absorption bands in the range of 400 to 1000 nm. The XRD results of the PtQDC shown in Fig. 1C were compared with those of PDF card PDF#87-0646, revealing that the crystal structure of PtQDC is identical to that of pure platinum. The XPS survey spectrum and high-resolution Pt 4f spectrum (Fig. 1D-E) indicate that the constituent element of PtQDC is platinum, which primarily exists in the zero-valent state. Lesion tissue fluid from EMS model mice was selected as the simulated environmental fluid to study the in vivo effects of PtQDC. As shown in Fig. 1F, the ectopic lesions from the model mice were excised, minced, ground, and then filtered with PBS to obtain the lesion tissue fluid. A schematic diagram illustrating the persistence and dissociation of the PtQDCs in the lesion tissue fluid is shown in Fig. 1G. The PtQDC existed first as intact clusters, which gradually underwent edge dissociation over time, subsequently releasing small, thin quantum dot clusters that collectively function within the lesion. As shown in Fig. 1H, the zeta potential of the retained PtQDC in the lesion tissue fluid was measured over time, with an initial value of −10.9 ± 1.6 mV. By day 3, the zeta potential decreased to −15.6 ± 1.4 mV before further decreasing to −20.2 ± 0.5 mV on day 7. The increase in the magnitude of the negative charge aligns with the larger specific surface area resulting from the dissociation of the PtQDCs. The fluorescence of the quantum dots released by PtQDC was observed with a UV lamp, and only the lesion tissue fluid containing PtQDC clearly exhibited blue–green fluorescence, as shown in Fig. 1I. In addition, the fluorescence spectrum of PtQDC shows that its maximum emission wavelength is 465 nm with laser excitation at 370 nm (Fig. 1J). STEM and mapping modes were employed to examine PtQDC samples retained in lesion tissue fluid for varying durations, as shown in Fig. 1K-M and Fig. S2, which revealed that PtQDC undergoes a gradual dissociation process. Moreover, HAADF imaging showed significant thinning of the sample, a reduction in edge sealing integrity, and the gradual emergence of small quantum dot clusters in the surrounding area, which ultimately transform into small Pt quantum dot clusters. STEM and mapping were conducted on the free quantum dots in the sample, and the results in Fig. 1N show that these species were Pt quantum dots with a size of 2–4 nm. This dissociation does not establish biodegradation or systemic clearance, and the ultimate fate, excretion pathways, and long-term safety of the released Pt quantum dots remain to be further determined.

**Fig. 1:**
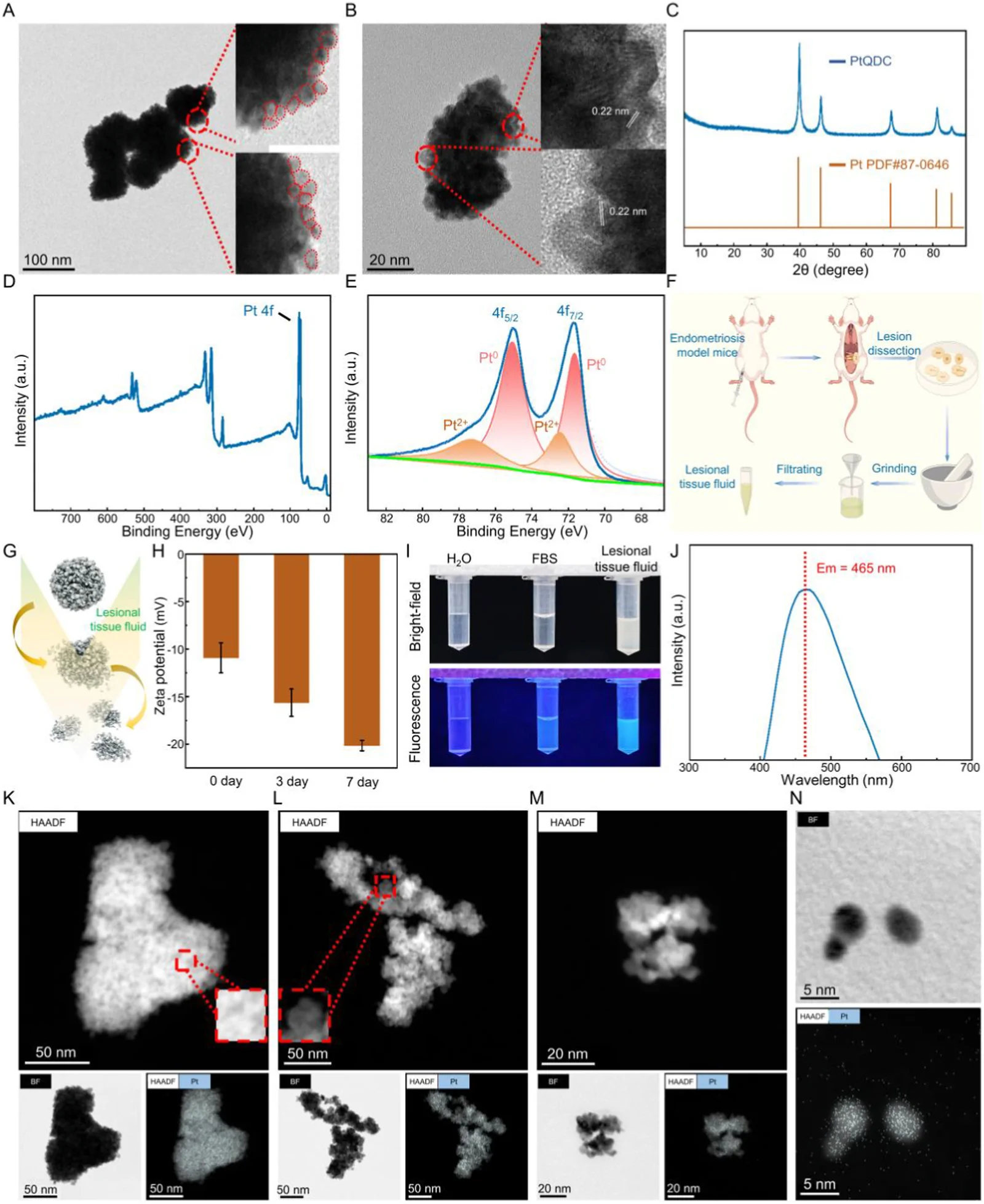
Characterization of PtQDC. (A) TEM and local magnification images of PtQDC. (B) High-resolution TEM image and lattice spacing results of PtQDC. (C) XRD results of PtQDC and Pt PDF#87-0646. (D) XPS results of PtQDC. (E) Fine XPS spectrum of PtQDC. (F) Schematic diagram of the preparation process of lesional tissue fluid. (G) Schematic diagram of the dissociation and release of Pt quantum dots from PtQDC in lesional tissue fluid. (H) Zeta potential results of PtQDC at different times in lesional tissue fluid. (I) Bright-field and fluorescence images of PtQDC in different solutions. (J) Fluorescence of the released Pt quantum dots upon excitation at 370 nm. (K)–(M) STEM and mapping results of PtQDC in the lesion tissue fluid after (K) 0 days, (L) 3 days and (M) 7 days. (N) STEM and mapping results of released Pt quantum dots.

### 2.2 Nanozyme activity of PtQDC

To investigate the free radical scavenging ability of PtQDC as a nanozyme, H_2_O_2_ and DPPH were selected as representative oxidant and nitrogen-centered radical models, respectively, to evaluate the concentration- and time-dependent scavenging activity of PtQDC, as shown in Fig. 2A–D. The UV absorption spectra of the DPPH and H_2_O_2_ solutions demonstrated that increasing the concentration of PtQDC or prolonging the treatment time could significantly increase the DPPH and H_2_O_2_ scavenging efficiency. To evaluate the stability of the enzymatic activity of PtQDC, the DPPH and H_2_O_2_ scavenging experiments were repeated. Each free radical was scavenged over four cycles, and the degree of scavenging at each time point in each cycle was statistically analyzed. As shown in Fig. 2E-H, the ability of PtQDC to scavenge the ROS and RNS was excellent, with the final scavenging efficiency in each cycle being almost the same and the clearance degree at each time point in each round being consistent. To verify the ability of PtQDC to scavenge multiple free radicals, ESR was used to evaluate the PTIO·, singlet oxygen (^1^O_2_), superoxide anion (O_2_^−^), and hydroxyl radical (·OH) scavenging ability of PtQDC. The results in Fig. 2I-L shows that PtQDC had a significant scavenging effect on the above four types of free radicals, which indicates that the enzymatic activity of PtQDC is highly efficient against a wide range of common free radicals in lesions. In addition, the photographs of PtQDC retained in PBS, culture medium and lesion tissue fluid for different periods demonstrate the good stability and biocompatibility of PtQDC in various solutions (Fig. 2M).

**Fig. 2:**
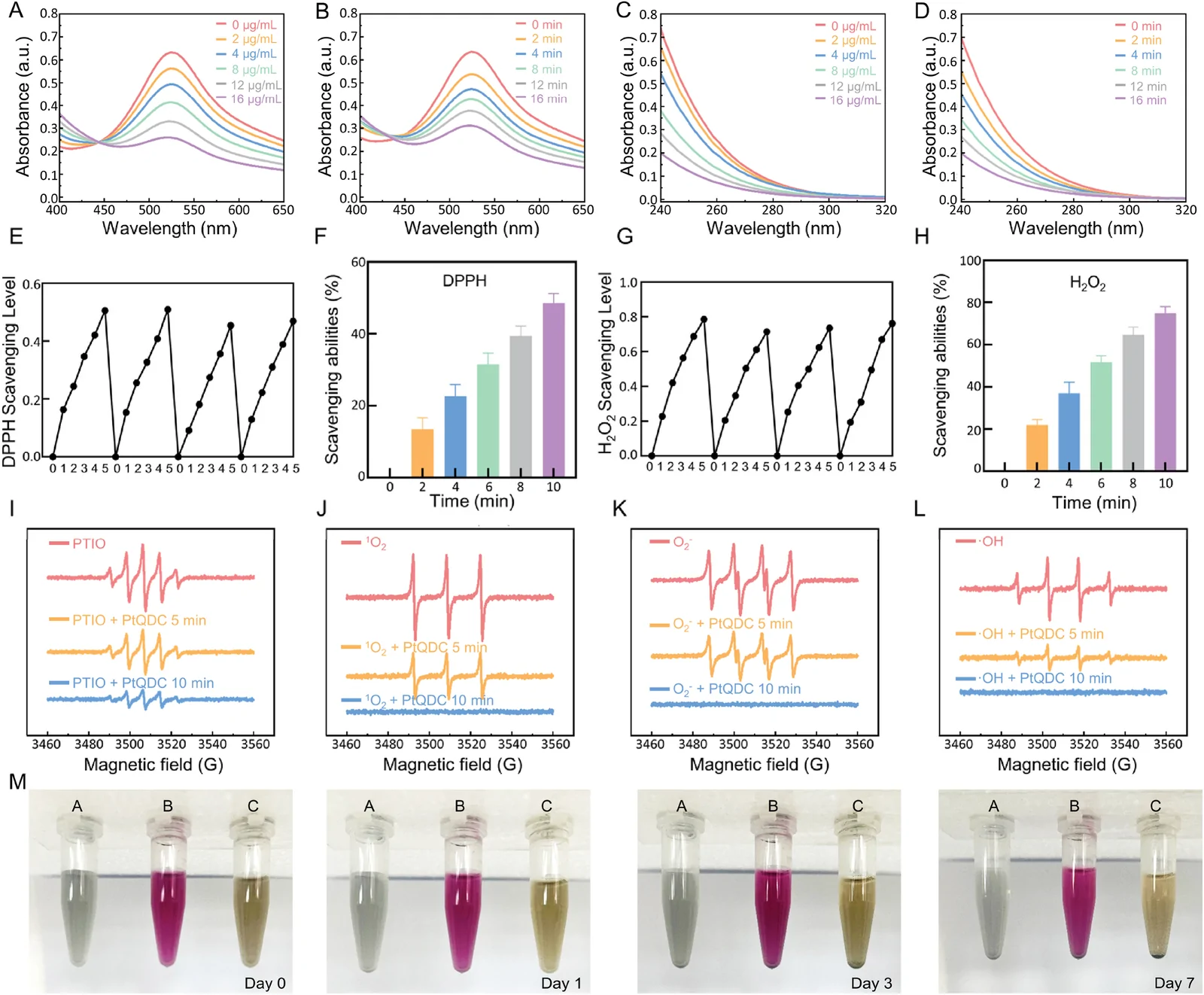
Nanozyme activity of PtQDC. (A) Absorption spectra of DPPH solutions treated with different concentrations of PtQDC. (B) Absorption spectra of a DPPH solution treated with PtQDC from 0 to 16 min. (C) Absorption spectra of H_2_O_2_ solutions treated with different concentrations of PtQDC. (D) Absorption spectra of a H_2_O_2_ solution treated with PtQDC from 0 to 16 min. (E) Stability of DPPH scavenging by PtQDC (4 cycles). (F) DPPH scavenging ability of PtQDC. (G) Stability of H_2_O_2_ scavenging by PtQDC (4 cycles). (H) H_2_O_2_ scavenging ability of PtQDC. (I) ESR spectra of PTIO· radicals in various groups. (J) ESR spectra of ^1^O_2_ radicals in various groups. (K) ESR spectra of O^2−^ radicals in various groups. (L) ESR spectra of ·OH radicals in various groups. (M) Stability of PtQDC after 0, 1, 3 and 7 days in various solutions. Group A, PBS. Group B, culture medium. Group C, lesional tissue fluid.

### 2.3 PtQDC-induced mitochondrial dysfunction and apoptosis in 12Z cells

PtQDC exploited the redox vulnerability of 12Z endometriotic epithelial cells by driving a concentration-dependent shift from their highly oxidative state toward a reductive state, thereby progressively reducing cell viability. Cell viability remained close to the control level at 25 μg/mL but decreased significantly at 50–200 μg/mL, with the greatest reduction observed at 200 μg/mL (Fig. 3A). This loss of viability was accompanied by a marked increase in GPX4 activity, which rose across the concentration range and reached similarly high levels at 100 and 200 μg/mL (Fig. 3B). DCFH-DA fluorescence concurrently weakened as the PtQDC concentration increased, demonstrating a progressive reduction in intracellular ROS (Fig. 3C). C11-BODIPY results further revealed a pronounced shift from the oxidized green-emitting state to the reduced red-emitting state, with the oxidized signal becoming nearly undetectable at the highest concentration (Fig. 3D). Consistent with this, the GSH/GSSG ratio increased sharply and plateaued at 100–200 μg/mL (Fig. 3E). Together, these changes show that PtQDC reinforced the GPX4–glutathione antioxidant system and effectively suppressed both intracellular ROS accumulation and lipid oxidation.

**Fig. 3:**
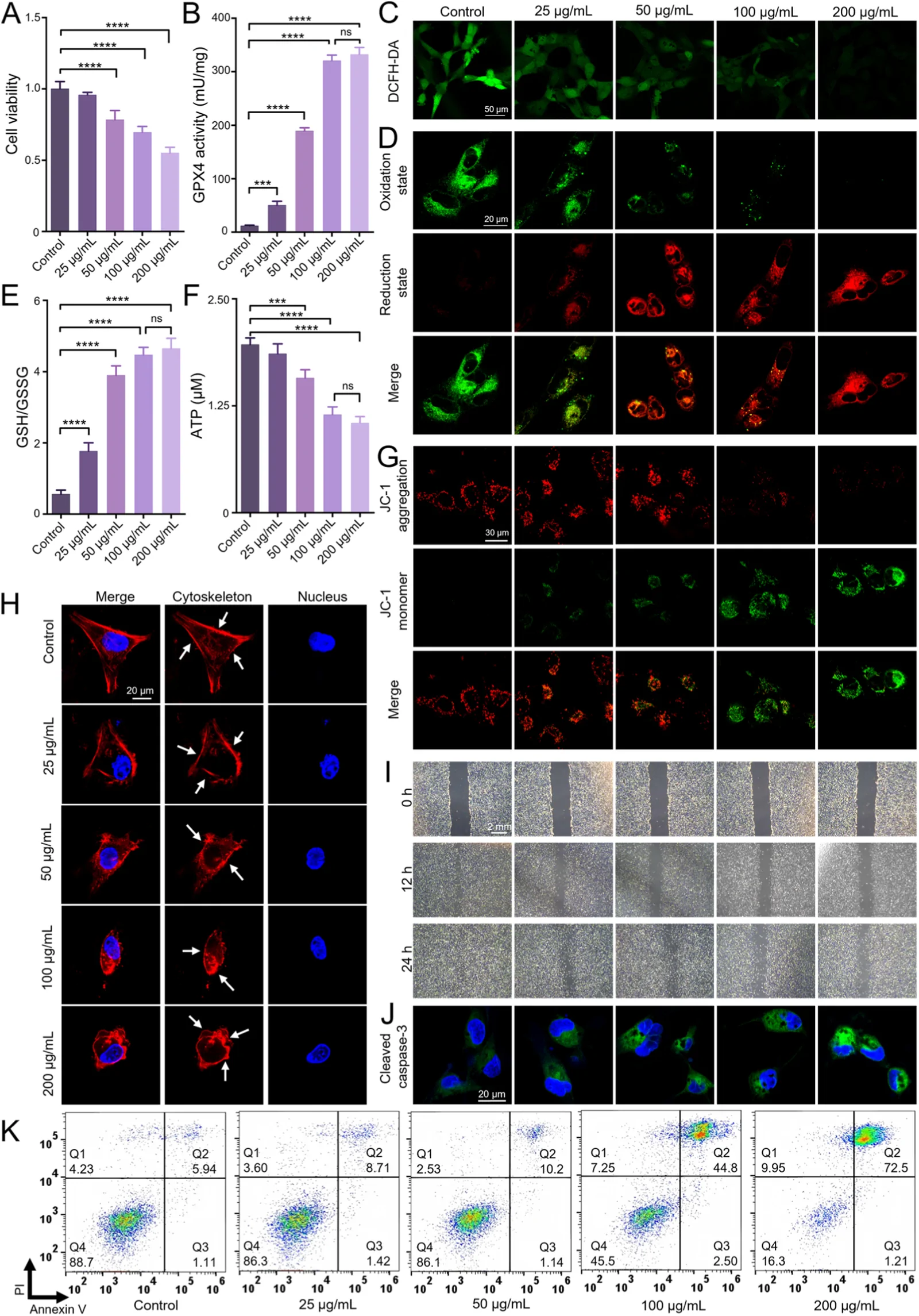
**PtQDC suppresses oxidative stress in 12Z cells.** (A) Viability of 12Z cells treated with PtQDC at the different concentrations. (B) Intracellular GPX4 activity in 12Z cells treated with PtQDC at the different concentrations. (C) DCFH-DA fluorescence images showing intracellular ROS levels. Scale bars, 50 μm. (D) C11-BODIPY 581/591 fluorescence images showing the oxidized (green) and reduced (red) states of 12Z cells treated with PtQDC at the different concentrations. Scale bars, 20 μm. (E) Intracellular GSH/GSSG ratio in 12Z cells treated with PtQDC at the different concentrations. (F) Intracellular ATP levels in 12Z cells treated with PtQDC at the different concentrations. (G) JC-1 fluorescence images showing JC-1 aggregates (red) and monomers (green) of 12Z cells in different groups. Scale bars, 30 μm. (H) Fluorescence images of the actin cytoskeleton of 12Z cells in different groups. Scale bars, 20 μm. (I) Images of the scratch test of treated 12Z cells at 0, 12, and 24 h. Scale bars, 2 mm. (J) Fluorescence images of the cleaved caspase-3 of 12Z cells in different groups. Scale bars, 20 μm. (K) Annexin V/PI flow-cytometric analysis of the apoptosis level in treated 12Z cells.

This PtQDC-induced reductive shift propagated into metabolic, mitochondrial, and structural dysfunction. Intracellular ATP declined progressively with increasing PtQDC concentrations and remained similarly low at 100 and 200 μg/mL (Fig. 3F), indicating that disruption of the oxidative state compromised cellular energy metabolism. JC-1 staining showed a parallel loss of red JC-1 aggregates and accumulation of green monomers, particularly at the two highest concentrations, demonstrating dissipation of the mitochondrial membrane potential (Fig. 3G). Whereas control cells exhibited an extended morphology with well-organized actin fibers, treated cells progressively lost their elongated architecture, displayed condensed or fragmented actin structures, and adopted a rounded morphology as the concentration increased (Fig. 3H).

The functional consequence of this response was reflected by suppressed migration and activation of apoptosis. In the wound-healing assay, control cells efficiently repopulated the scratched area over 12–24 h, whereas PtQDC progressively impaired wound closure, with the strongest inhibition occurring at 100 and 200 μg/mL (Fig. 3I). Cleaved caspase-3 fluorescence also increased across the concentration series, supporting activation of an apoptotic execution program (Fig. 3J). This response was corroborated by Annexin V/PI flow cytometry: the combined Annexin V-positive population increased from approximately 7.1% in control cells to 47.3% and 73.7% after treatment with 100 and 200 μg/mL PtQDC, respectively, with a marked accumulation of Annexin V/PI double-positive late apoptotic cells (Fig. 3K). Together, these results indicate that PtQDC-induced reductive stress disrupts mitochondrial function and promotes apoptosis in 12Z cells.

### 2.4 In vivo treatment of EMS mediated by PtQDC

The inhibitory effect of PtQDC on lesion growth was systematically evaluated in vivo in a mouse EMS model. The experimental procedure is shown in Fig. 4A. Endometrial tissues from donor mice were first transplanted into recipient mice to construct the model [33], and then, PBS was given as a model control or low-dose PtQDC (PtQDC-L) or high-dose PtQDC (PtQDC-H) were administered for treatment. All the model groups produced lesions, except for the sham-operated group (CON), indicating successful model construction with lesion growth and pathological changes in the abdominal cavity (Fig. 4B). Moreover, the pathological conditions of the two groups of mice treated with PtQDC significantly improved, as indicated by significant decreases in the number and volume of the intra-abdominal lesions after PtQDC treatment (Fig. 4C-E). PtQDC treatment significantly reduced uterine adhesion scores, indicating alleviation of lesion-associated adhesions during the treatment period (Fig. 4F). In vivo experiments demonstrated that PtQDC treatment could effectively inhibit the growth of endometriotic lesions, attenuate the associated pathological changes, and have potential therapeutic effects on EMS. Meanwhile, the treatment approach of PtQDC demonstrates effective biological safety during the therapy period (Fig. S3–S4).

**Fig. 4:**
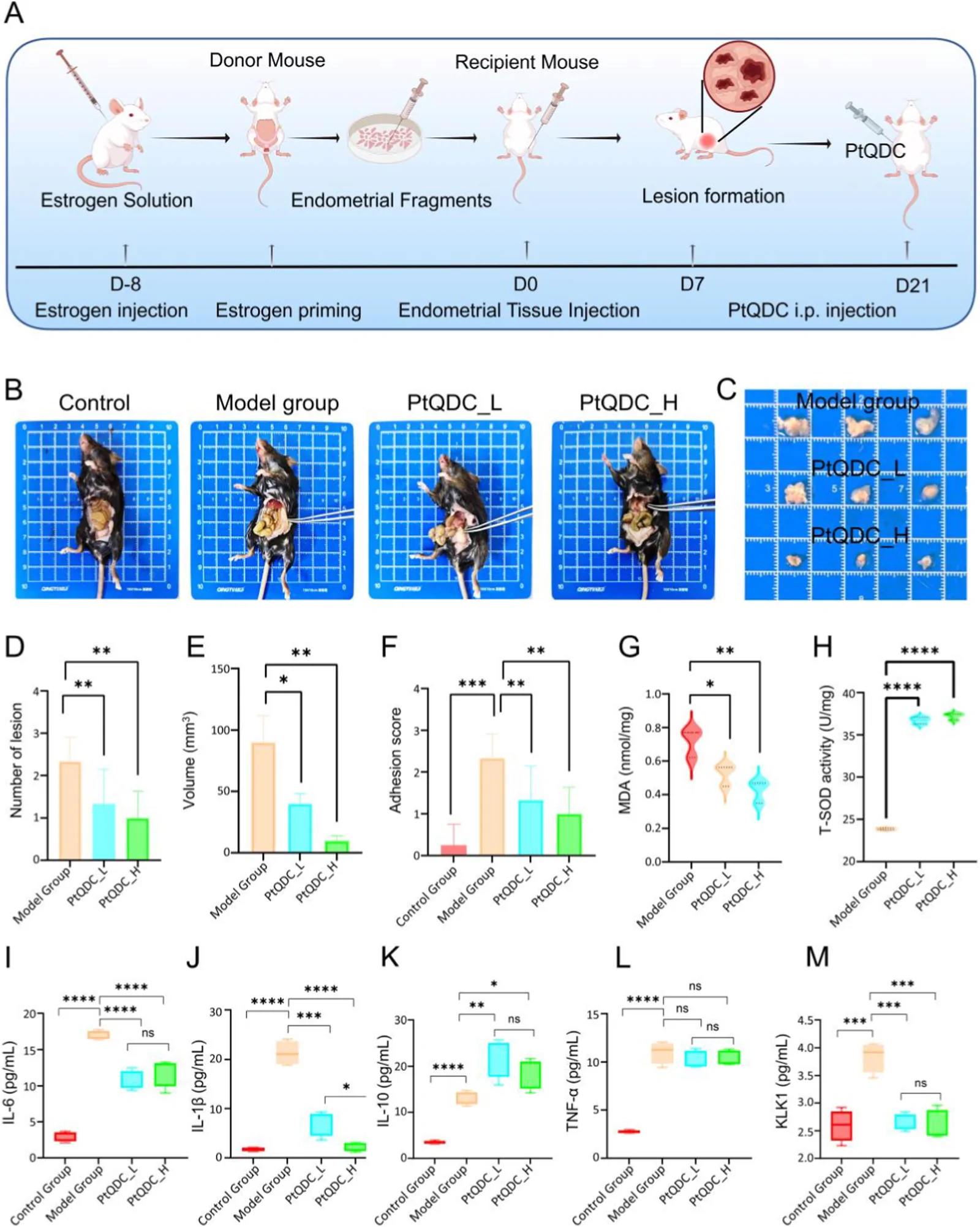
**In vivo treatment of EMS mediated by PtQDC**. (A) Flowchart of the experimental design showing the construction of the mouse EMS model and the PtQDC treatment process. (B) Photographs of representative mice in different treatment groups (sham operation group (CON), model group (Model), low-dose PtQDC group (PtQDC-L), and high-dose PtQDC group (PtQDC-H)). In each group, n = 3. (C) Representative lesion diagrams of each group. (D–F) Evaluation of endometriotic foci in the abdominal cavity: (D) statistical plot of the number of foci in each group; (E) statistical plot of the volume of foci in each group; and (F) statistical plot of the uterine adhesion scores. (G-H) Analysis of oxidative stress indicators in lesion tissues: (G) statistical plot of malondialdehyde (MDA) levels; and (H) statistical plot of superoxide dismutase (SOD) activity. (I–M) Detection of inflammation- and pain-associated mediators in abdominal lavage fluid: (I) IL-6, (J) IL-1β, (K) IL-10, (L) TNF-α and (M) KLK1. Data are presented as the mean ± SD. *p < 0.05, **p < 0.01, ***p < 0.001, and ****p < 0.0001; ns, not significant.

Oxidative stress plays a key role in the occurrence and development of EMS by promoting the survival, proliferation, inflammatory response, and fibrosis of ectopic lesions through multiple mechanisms [34], [35]. After PtQDC treatment, MDA levels significantly decreased, whereas SOD levels significantly increased, indicating that PtQDC alleviated oxidative stress in the lesions (Fig. 4G–H).

To evaluate the effect of PtQDC on the abdominal inflammatory microenvironment and pain-associated signaling, inflammatory mediators and KLK1 in abdominal lavage fluid were measured by ELISA. Compared with the control group, the model group exhibited significant increases in IL-6, IL-1β, TNF-α, and KLK1, together with a significant increase in IL-10, indicating pronounced inflammation accompanied by enhanced KLK1-associated signaling. Compared with the model group, PtQDC treatment, particularly at the high dose, reduced IL-6, IL-1β, and KLK1 while increasing IL-10, whereas TNF-α remained unchanged (Fig. 4I–M). Collectively, these findings suggest that PtQDC remodeled the inflammatory peritoneal microenvironment and concurrently attenuated pain-associated signaling. Future studies could assess the durability of these therapeutic benefits and lesion recurrence following treatment withdrawal.

### 2.5 PtQDC-mediated reprogramming of the EMS microenvironment

In the present study, the direct regulatory effect of PtQDC on the local inflammatory microenvironment of endometriotic lesions was visually assessed at the tissue level by immunofluorescence staining. The results clearly demonstrated that PtQDC treatment effectively remodeled the immune balance of the lesion, resulting in strong bidirectional immunoregulation. Compared with that in the untreated model group, the fluorescence intensity of the key proinflammatory factors TNF-α, IL-1β and IL-6 in the lesion tissues significantly decreased in a dose-dependent manner after PtQDC treatment (Fig. 5A–E). These results confirm that PtQDC can not only reduce the concentration of inflammatory factors in peritoneal lavage fluid but also act directly on the lesion tissue itself, effectively curbing the vicious proinflammatory cycle. More importantly, the fluorescence intensity of the anti-inflammatory factor IL-10 was significantly increased in the PtQDC-treated groups. These findings suggest that the therapeutic effect of PtQDC is not simply anti-inflammatory but rather a more precise immune rebalancing effect. While PtQDC suppresses harmful inflammation, it actively promotes the expression of anti-inflammatory factors with protective effects, which help guide the microenvironment toward tissue repair and homeostasis restoration.

**Fig. 5:**
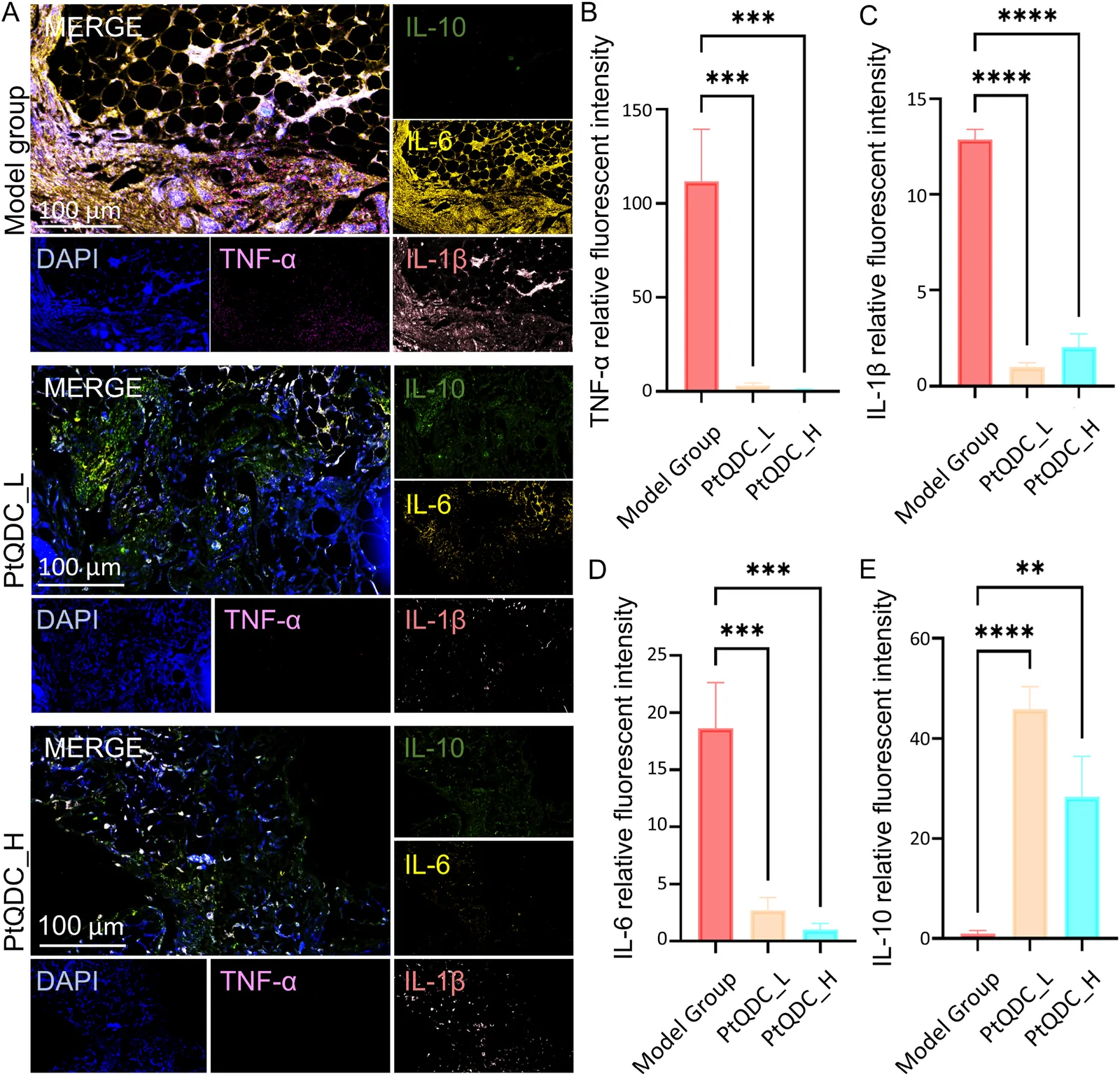
**Effect of PtQDC treatment on the expression of inflammatory factors in endometriotic lesions.** (A) Representative immunofluorescence staining images of endometriotic lesion tissues. From top to bottom are the model group (Model), the low-dose PtQDC-treated group (PtQDC-L) and the high-dose PtQDC-treated group (PtQDC-H). The fluorescence signals of the anti-inflammatory factor interleukin-10 (IL-10, green), the proinflammatory factor interleukin-6 (IL-6, yellow), the proinflammatory factor tumor necrosis factor-α (TNF-α, purple), and the proinflammatory factor interleukin-1β (IL-1β, pink). Scale bar: 100 μm. (B–E) Semiquantitative statistical analysis of the expression levels of the following factors related to inflammation in focal tissues: (B) TNF-α, (C) IL-1β, (D) IL-6 and (E) IL-10. n = 3.

To evaluate how PtQDC affects angiogenesis and the hypoxic environment, as shown in Fig. 6A and Fig. 6C–D, both VEGF and the endothelial marker CD31, were shown to have strong fluorescent signals in the lesion tissues of the Model group, indicating the presence of active pathological angiogenesis in the lesions. After PtQDC intervention, both the VEGF and CD31 fluorescence signals were significantly attenuated in a dose-dependent manner, with the most significant inhibitory effect observed in the PtQDC-H group. Moreover, the fluorescence intensity of hypoxia-inducible factor-1α significantly decreased after PtQDC treatment (Fig. 6B and Fig. S5). CD10, an endometrial mesenchymal-specific marker, was detected in all the lesions of each group, confirming the successful and consistent construction of the EMS model (Fig. S6). These results reveal another key mechanism by which PtQDC inhibits the growth of endometriotic lesions: by improving local hypoxia and inhibiting pathological angiogenesis upstream. The high coexpression of VEGF, a major proangiogenic factor, and CD31, a vascular endothelial cell-specific marker, is direct evidence of exuberant neovascularization in the lesions. The concurrent reductions in VEGF and CD31 signals support the inhibitory effect of PtQDC on pathological angiogenesis in the lesions. Angiogenesis supports the survival and growth of endometriotic lesions by supplying oxygen and nutrients. Therefore, the inhibition of pathological angiogenesis may restrict lesion growth and contribute to the therapeutic effect of PtQDC.

**Fig. 6:**
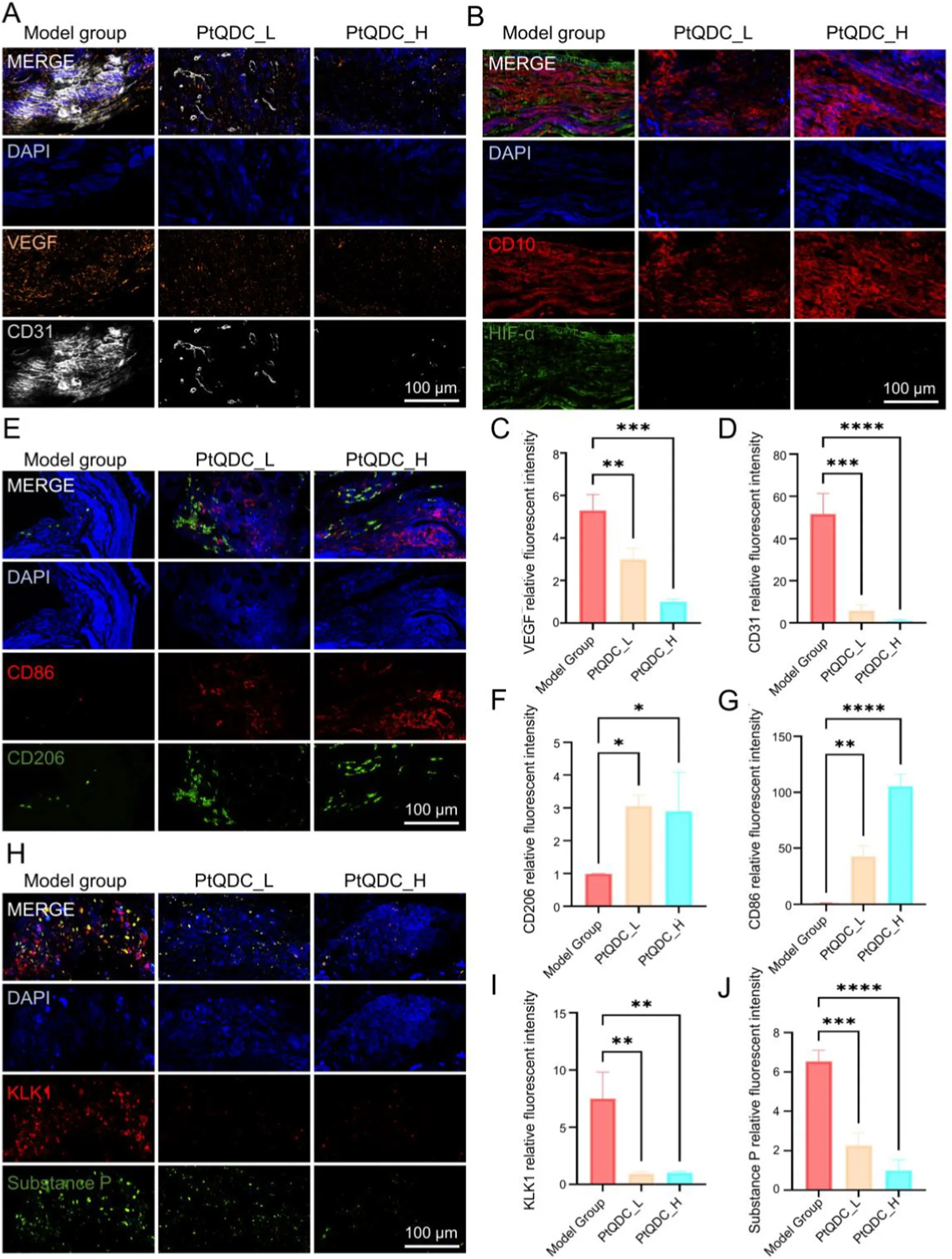
**Effects of PtQDC on angiogenesis, the immune microenvironment and pain-associated signaling in vivo.** (A) Representative immunofluorescence staining images of vascular endothelial growth factor (VEGF) and the vascular endothelial marker CD31. (B) Representative immunofluorescence staining images of focal tissue hypoxia-inducible factor-1α (HIF-1α) and the endometrial mesenchymal marker CD10. (C) – (D) Statistical analysis of the relative fluorescence intensity of (C) VEGFA and (D) CD31. n = 3. (E) Representative staining images of the M1 macrophage marker CD86 and the M2 macrophage marker CD206. (F) – (G) Statistical analysis of the relative fluorescence intensity of (F) CD206 and (G) CD86. n = 3. (H) Representative immunofluorescence staining images of pain-related neuropeptide substance P and kinin-releasing enzyme KLK1 in lesion tissue. (I) – (J) Statistical analysis of the relative fluorescence intensity of (I) KLK1 and (J) Substance P. n = 3.

To investigate the effect of PtQDC on the local immune microenvironment, the expression of the macrophage-associated markers CD86 and CD206 in lesion tissues was examined by immunofluorescence staining. As shown in Fig. 6E–G, both CD86 and CD206 signals were weak in the untreated model group but increased after PtQDC treatment, indicating substantial changes in macrophage-associated marker profiles within the lesions. The increase in CD86 was more pronounced in the PtQDC-H group, suggesting a greater shift toward an M1-associated phenotype at the higher dose. Previous studies have shown that M2 macrophages may promote the invasion and migration of ectopic endometrial cells while contributing to tissue remodeling and fibrosis [36], whereas M1-associated activity may restrict lesion progression [37]. Thus, the PtQDC-induced changes in macrophage marker profiles may contribute to remodeling of the lesion immune microenvironment.

Pain is a major clinical manifestation of EMS. To determine whether PtQDC affects pain-associated molecular signaling, the expression and spatial distribution of KLK1 and substance P in endometriotic lesions were examined by immunofluorescence staining (Fig. 6H–J). PtQDC treatment reduced both KLK1 and substance P expression compared with the model group, with more pronounced reductions in the PtQDC-H group. The strong overlapping fluorescence observed in the model group was also markedly weakened following PtQDC treatment, indicating reduced abundance and spatial overlap of these mediators within the lesions. Because the kallikrein–kinin system and substance P contribute to nociceptive sensitization and neurogenic inflammation, their concurrent reduction suggests that PtQDC may attenuate pain-associated molecular signaling. Together, these findings indicate that PtQDC alleviates lesional inflammation while modulating molecular pathways associated with EMS-related pain.

### 2.6 Transcriptomic profiling of multidimensional PtQDC therapeutic mechanisms

The preceding results showed that PtQDC modulated oxidative stress, inflammation, and angiogenesis, whereas the molecular changes associated with these effects remained unclear. RNA-seq was therefore performed to characterize the transcriptomic response to PtQDC treatment. A total of 108 differentially expressed genes between the PtQDC treatment group and the model group were identified on the basis of |log_2_FC| ≥ 1 and adjusted p < 0.05 (Fig. 7A and 7B**)**. A total of 254 significantly enriched GO terms were identified, which focuses on biological processes, cellular components, and molecular functions on the basis of the P value, and the top 10 terms are shown in Fig. 7C. Inflammation-related pathways, including neutrophil chemotaxis, acute inflammatory response to antigenic stimulus, CCR chemokine receptor binding, oxidoreductase activity, lipid metabolic processes, NAD binding, and oxidative stress-related pathways, as well as cell adhesion, extracellular matrix, and collagen-containing extracellular matrix and adhesion pathways, can directly lead to fibrosis and adhesion of the lesion and trigger EMS pain and infertility owing to abnormal matrix deposition and remodeling. The results of the KEGG analysis were highly compatible with the results of the GO analysis (Figs. S7–S9). In terms of cell adhesion, ECM-receptor interactions, cell adhesion molecules, and focal adhesions are the molecular basis of the initiation and invasiveness of EMS, and these three pathways can directly regulate cytoskeletal reorganization, provide an impetus for cell migration, and transmit powerful proliferative and antiapoptotic signals, which further induce fibrosis [38]. From the inflammation–immune perspective, three pathways, cytokine‒cytokine receptor interactions, chemokine signaling pathways, and viral protein interactions with cytokines and chemokines, constitute a complex intercellular communication network. The binding of cytokines and chemokines to their receptors activates downstream inflammatory signaling, creating the chronic inflammatory environment that is characteristic of EMS. Moreover, from a metabolic perspective, the metabolism of xenobiotics by cytochrome P450s, tyrosine metabolism, fatty acid degradation and other pathways can mediate oxidative stress damage, and ROS can cause DNA damage and lipid peroxidation, further exacerbating inflammation and cellular damage. Correspondingly, metabolic reprogramming that occurs in ectopic cells as a survival adaptation increases intracellular ROS levels, which can further exacerbate oxidative damage [39]**.** In addition, among the top 20 enriched pathways, the PI3K-Akt signaling pathway, which is continuously activated in EMS tissue to help ectopic cells resist apoptosis, promote proliferation, and induce neovascularization, is the most critical signaling hub for the maintenance and growth of lesions [40]. The role of the renin-angiotensin system also warrants attention. Angiotensin II, which is highly expressed in endometriotic lesions, potently promotes neovascularization, fibrosis, and inflammation and is involved in the regulation of pain signaling, another important pathway that links inflammation, angiogenesis, and fibrosis [41]. Estrogen promotes the growth, inflammation, and angiogenesis of endometriotic lesions [42]. The lack of a significant difference in this pathway in the current analysis may be related to the relatively limited sequencing sample size; in subsequent studies, we will expand the sample size and conduct targeted validation analyses for steroid synthases localized to the lesion.

**Fig. 7:**
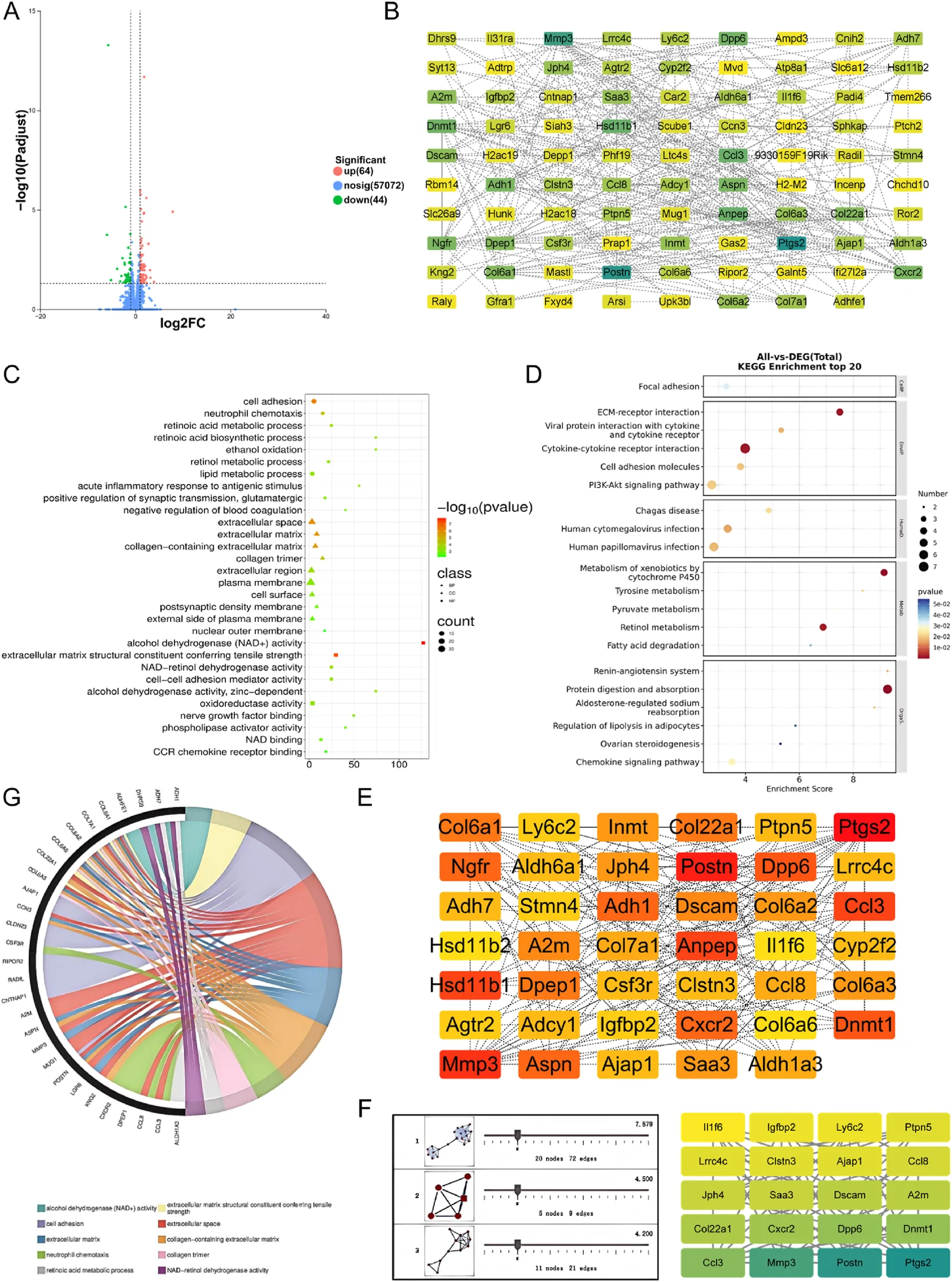
**Bioinformatics analysis of PtQDC-treated endometriotic tissue**. (A) Volcano plot of differentially expressed genes (DEGs). The differentially expressed genes are indicated by the log_2_FC (fold change) on the horizontal axis and -log_10_(Padj) on the vertical axis. Red dots represent upregulated genes (log_2_FC ≥ 1 and Padj < 0.05), green dots represent downregulated genes (log_2_FC ≤ −1 and adjusted Padj < 0.05), and blue dots represent genes whose expression did not significantly differ. (B) PPI network of significantly differentially expressed genes. (C) Bubble diagram of the results of the GO functional enrichment analysis of the differentially expressed genes. The top 10 most significantly enriched GO terms in the biological process (BP), cellular component (CC) and molecular function (MF) categories are shown. (D) Bubble plot of the top 20 enriched KEGG pathways. The pathway names are presented on the vertical axis, and the enrichment factors are shown along the horizontal axis. The bubble size represents the number of genes enriched in the pathway, and the bubble color represents significance of the enrichment. (E) Interaction network of the 41 core targets; the darker the color is, the higher its degree value is. (F) MCODE analysis of the PPI network, showing the three identified modules and the highest-scoring module. (G) GO term enrichment chord diagram.

### 2.7 PtQDC-mediated regulation of core pathogenic genes

To gain a deeper understanding of the differentially expressed genes identified by RNA-seq, a STRING protein–protein interaction network was constructed with the Hubba plugin, and 41 genes were defined as core genes on the basis of their degree values being greater than average. The degree values of Ptgs2, Postn, and Mmp3 were the highest, at 25, 24, and 22, respectively, as shown in Fig. 7E. When the MCODE algorithm was used to search for the densely connected regions of 108 genes, three gene clusters were identified, and their MCODE scores were calculated (Fig. 7F), with a higher score indicating a closer connection within the cluster. The MCODE score of the core cluster containing Ptgs2, Postn, and Mmp3 was 7.579, once again proving the central roles of these three genes. Furthermore, the functions of these three genes were analyzed in Fig. 7G. MMP3 degrades extracellular matrix components and is a key enzyme involved in tissue remodeling and cell invasion, and the main pathways associated with MMP3 are ECM–receptor interactions, focal adhesion, and cytokine–cytokine receptor interactions. Moreover, MMP3 expression is a hallmark of the invasive ability of endometriotic lesions, as it acts as a “molecular scissor” to help ectopic cells “cut” and invade the stroma of the peritoneal cavity or other organ surfaces; additionally, MMP3 is a key player in the establishment of foci and deep infiltration [43]. PTGS2 catalyzes the synthesis of prostaglandins from arachidonic acid and is associated with the cytokine‒cytokine receptor interaction pathway, chemokine signaling pathway, and angiogenesis-related pathway. PTGS2 is a central driver of inflammation and pain, and its involvement in the production of prostaglandins directly leads to local vasodilatation, edema, and pain sensitization and promotes lesion proliferation and neovascularization; moreover, PTGS2 is a key molecular target of chronic pelvic pain associated with EMS [44]. In contrast, POSTN, an extracellular matrix protein, mediates cell‒matrix adhesion and promotes fibrosis through ECM‒receptor interactions and the PI3K‒Akt signaling pathway. POSTN is a key mediator of fibrosis and tissue remodeling, as it both assists ectopic cells in firmly adhering to the ECM and facilitates their colonization by transmitting survival signals and by driving fibrotic progression around foci, leading to the formation of adhesions [45]. Therefore, the entire process of EMS development, from lesion implantation, inflammation development, and fibrosis formation, can be completely linked by these three genes.

Moreover, the expression of these genes was further examined in a publicly available dataset comprising 408 clinical samples, including control in situ endothelium, EMS in situ endothelium, control peritoneum, patient peritoneum, peritoneal foci, deep infiltrating foci, and ovarian cystic foci [38], [46]. Compared with that in normal endothelial tissues, the expression of PTGS2, POSTN, and MMP3 was found to be elevated in all foci, especially in deep infiltrating foci and ovarian foci, which demonstrated that there is a direct clinical correlation between the expression of these three genes and the development of EMS; furthermore, the negative regulation of these three genes may help to control the development and progression of EMS in the clinic (Fig. 8A-C). PtQDC also inhibited these three factors in vivo, with the downregulation of MMP3, Postn, and Ptgs2 in lesion tissues (Fig. 8D). To further explore the associations between the 41 core genes and disease stage, gene expression data from 26 patients associated with minimal/mild EMS and 48 patients associated with moderate/severe EMS was evaluated [47], among which only ALDH1A3 expression was significantly elevated in tissue from patients with severe EMS (Fig. 8E-F). PtQDC significantly downregulated the expression of ALDH1A3, which is involved mainly in the retinol metabolic pathway, which also includes several other genes, including ADH and RDH (Fig. 8G). The terms “retinoic acid metabolic process”, “retinol biosynthetic process”, and “retinol metabolic process” were also highly enriched according to the results of the GO analysis, demonstrating the possible critical role of vitamin A in EMS. Vitamin A regulates the normal differentiation of and cyclic changes in endometrial cells. Abnormalities in vitamin A signaling may be associated with the development of EMs. Moreover, vitamin A, an important immunomodulator, can alter the functions of macrophages and T cells. Chronic inflammation, as a core feature of EMS, alters immunity and thus the inflammatory state and is also a current research hotspot [48], [49]**.** This result, in turn, provides a new research direction to study the use of PtQDC for EMS treatment (Fig. 8H).

**Fig. 8:**
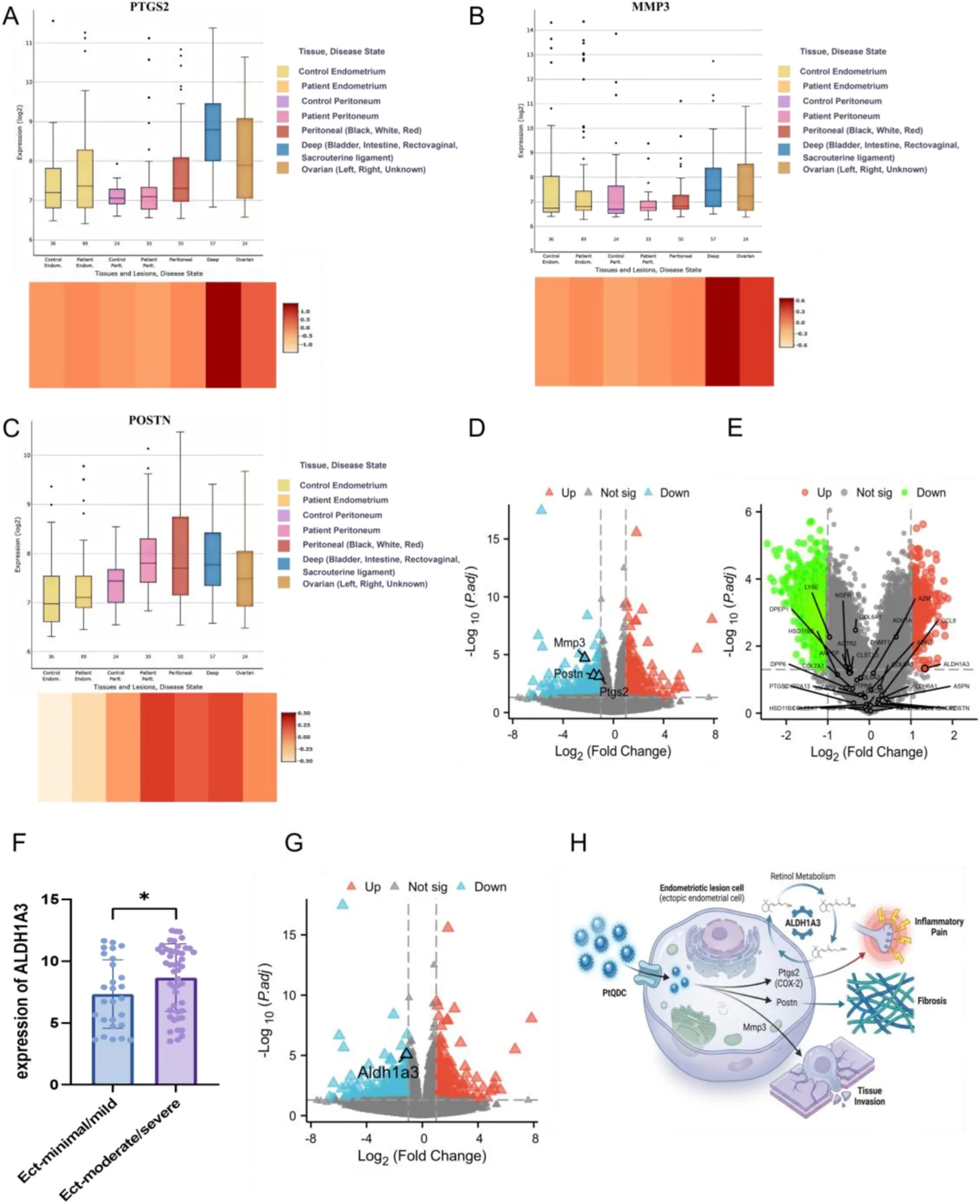
**Identification of core targets and bioinformatics analysis of PtQDC for the treatment of EMS**. (A–C) Correlation analysis of (A) PTGS2, (B) MMP3, and (C) POSTN expression in clinical samples. The heatmap below shows the normalized expression levels of the genes in the same set of samples, wherein red indicates high expression. (D) Volcano plot of genes differentially expressed after PtQDC treatment, highlighting Ptgs2, Mmp3 and Postn. (E) Validation of ALDH1A3 expression. (F) Histogram of the differential expression of the Aldh1a3 gene in minimal/mild vs moderate/severe. (G) Volcano plot of genes that were differentially expressed after PtQDC treatment, highlighting Aldh1a3. (H) Roles of PTGS2, MMP3, POSTN and ALDH1A3 in EMS disease progression.

## 3 Conclusion

In this study, we developed PtQDC as a dissociable nanozyme for the nonhormonal treatment of endometriosis. PtQDC exhibited gradual dissociation in lesion tissue fluid and sustained reactive species-scavenging activity. In 12Z endometriotic cells, this antioxidant activity shifted the highly oxidative cellular state beyond a favorable redox window, accompanied by impaired energy metabolism, mitochondrial dysfunction, and apoptosis, while showing favorable compatibility with HESC cells. In a mouse model of endometriosis, PtQDC reduced lesion number and volume, alleviated adhesion, and remodeled the peritoneal microenvironment by suppressing proinflammatory mediators, restoring IL-10, and altering macrophage marker profiles. PtQDC also inhibited pathological angiogenesis and attenuated pain-associated molecular signaling, as reflected by reduced VEGF, CD31, HIF-1α, KLK1, and substance P expression. Transcriptomic and bioinformatic analyses further showed that PtQDC downregulated Ptgs2, Mmp3, and Postn, supporting its effects on interconnected processes involving inflammation, tissue invasion, and fibrosis. Collectively, these findings establish PtQDC as a multitarget nanozyme strategy for modulating the complex lesion microenvironment and support its further preclinical evaluation as a nonhormonal therapy for endometriosis.

## 4 Statistical analysis

Quantitative data are presented as the mean ± SD. The two-tailed Student's t-test was used to identify any significant differences. All statistical differences were calculated by using GraphPad Prism 8.0. Values of P < 0.05 were statistically significant (*P < 0.05, **P < 0.01, ***P < 0.001, and ****P < 0.0001).

## CRediT authorship contribution statement

**Cheng Cao:** Writing – review & editing, Writing – original draft, Methodology, Investigation. **Jie Ding:** Writing – review & editing, Writing – original draft, Software, Resources. **Huifang Hao:** Validation, Resources, Methodology. **Yingao Jiao:** Supervision, Software. **Yu Cao:** Validation, Methodology. **Yanfei Fu:** Visualization, Validation. **Xinyuan Cui:** Visualization, Validation, Methodology. **Dengyao Gao:** Visualization, Supervision. **Yan Gao:** Visualization, Methodology. **You Wang:** Writing – review & editing, Data curation, Conceptualization. **Chaoqin Yu:** Funding acquisition, Conceptualization. **Ting Wang:** Writing – review & editing, Data curation, Conceptualization. **Yanlei Liu:** Project administration, Funding acquisition, Conceptualization.

## Ethics statement

All animal experiments were carried out at the Central Laboratory of Changhai Hospital and approved by the Experimental Animal Ethics Review Committee (Registration no. CHEC(AE) 2022-002).

## Declaration of competing interest

All authors declare no conflicts of interest.

## Data Availability

Data will be made available on request.
