# Supplementary material for: Bio-dissociable platinum quantum dot cluster nanozymes for nonhormonal endometriosis therapy through microenvironment remodeling
Source: Mater Today Bio. 2026 Oct 26;41:103785. doi: 10.1016/j.mtbio.2026.103785 (PMC13738020; doi:10.1016/j.mtbio.2026.103785)
Supplement: Supplementary file 1 [file mmc1.docx]

Supporting Information:

Bio-dissociable Platinum Quantum Dot Cluster Nanozymes for Nonhormonal Endometriosis Therapy through Microenvironment Remodeling

*Cheng Cao^Ϯ^,* *Jie Ding^Ϯ^,* *Huifang Hao, Yingao Jiao, Yu Cao, Yanfei Fu, Xinyuan Cui, Dengyao Gao, Yan Gao, You Wang,* Chaoqin Yu,* Ting Wang,* and Yanlei Liu**

Cheng Cao, Huifang Hao, Yingao Jiao, Yu Cao, Yanfei Fu, Xinyuan Cui, Dengyao Gao, Yan Gao, Yanlei Liu

Institute of Intelligent Health Diagnosis and Treatment, School of Automation and Intelligent Sensing, Shanghai Jiao Tong University, Shanghai 200240, People's Republic of China

E-mail: liuyanlei@sjtu.edu.cn

Jie Ding, Chaoqin Yu

Department of Traditional Chinese Medicine, The First Affiliated Hospital of Naval Medical University, Shanghai 200433, People's Republic of China

E-mail: chqyu81@163.com

Ting Wang

Obstetrics & Gynecology Hospital of Fudan University, Shanghai Key Lab of Reproduction and Development, Shanghai Key Lab of Female Reproductive Endocrine Related Diseases CN, Shanghai 200011, People's Republic of China

E-mail: wangting1771@fckyy.org.cn

You Wang

Shanghai Key Laboratory of Gynecologic Oncology, Renji Hospital, School of Medicine, Shanghai Jiao Tong University, Shanghai 200127, People's Republic of China

E-mail: wanghh0163@163.com

**Ϯ: The equal contribution to this article**

**Experimental Methods**

**Materials:** HCl, PtCl4, N,N-dimethylformamide (DMF) and H2O2 were purchased from Sigma‒Aldrich (Shanghai, China). Ultrapure water (18.2 MΩ/cm at 25°C) purified by a Milli-Q system was used throughout the experiments. The Cell Counting Kit-8, Living/Dead cell double staining kit, Reactive Oxygen Species assay kit, GPX4 assay kit, GSH/GSSG assay kit, ATP assay kit and Lipid Oxidation assay kit were obtained from Beyotime Institute of Biotechnology (China).

**Measurements and characterization:** A TALOS F200X transmission electron microscope (Thermo Fisher, USA) was used to characterize the morphology and size of the samples. A UNIC-3802 spectrophotometer was used to record the UV‒vis absorption spectra of the samples. XPS (Axis UltraDLD; Shimadzu, Japan) was used to determine the valence states of the PtQDC. An FLS1000 steady-transient fluorescence spectrometer (Edinburgh Instruments, UK) was used to determine the fluorescence excitation and emission spectra of the samples. Intracellular fluorescence was measured with a TCS SP8 STED 3X (Leica, China).

**Preparation of PtQDC**: Pt quantum dot clusters were synthesized by a one-step hydrothermal method. First, 64 mg of PtCl_4_ was added to a mixed solution of 10 mL of DMF and 20 mL of ethanol and thoroughly mixed, after which 3 mL of 3 M HCl solution was added. The above solution was placed in a reaction vessel and subjected to a hydrothermal reaction at 100°C for 24 hours. After natural cooling, the precipitate (PtQDC) was obtained by centrifugation.

**Biological dissociation of PtQDC**: EMS lesions were extracted from the abdominal cavities of EMs model mice, crushed, and filtered to obtain the lesion tissue fluid. After the incubation of PtQDC with this solution for varying durations, the samples were characterized via TEM and zeta potential measurements.

**Detection of free radical scavenging**: The ability of PtQDC to scavenge DPPH and H_2_O_2_ was examined by monitoring changes in the UV‒Vis absorption spectra in PBS at different times, and PTIO·, _1_O^2^, O^2-^, and ·OH radical scavenging by PtQDC was verified by ESR.

**Cell Culture and Processing**: The human EMs epithelioid cell line 12Z was selected as the lesion-mimicking cell line for the experiments, and human endometrial stromal cell line (HESCs) were used as normal control cells. All the cells were routinely allowed to adhere to the wall in an incubator at 37°C and 5% CO₂ using high-glucose DMEM (supplemented with 10% fetal bovine serum and 1% penicillin‒streptomycin solution). Cells were grown to the logarithmic growth phase for the experiments. A PtQDC stock solution was prepared in PBS and diluted with serum-free medium to the desired working concentration at the time of use. On the basis of the results of the preliminary experiments, 100 μg/mL PtQDC was selected as a suitable concentration, and the survival rate of HESC cells treated with this concentration of PtQDC was greater than 86%, indicating high compatibility with normal cells.

**Cell viability assay**: A CCK-8 assay was used to detect cell viability. 12Z cells were inoculated in 96-well plates at a density of 5×10³ per well and cultured for 12 h. After the cells had attached to the wall, the medium was replaced with medium containing different concentrations of PtQDC (0–400 μg/mL), and the cells were cultured for an additional 24 h. Afterward, 10 μL of CCK-8 solution was added to each well, and after 2 h of incubation, the absorbance values were measured at 450 nm with an enzyme marker. Cell viability was normalized to that of the control group.

**Assessment of cell migration ability**: The effect of PtQDC on the migration ability of 12Z cells was assessed using a cell scratch assay. Cells were inoculated in 6-well plates and grown to 90% confluence, after which a scratch was created in the center of the well plate with a 200 μL sterile pipette tip. After gently rinsing with PBS to remove detached cells, the medium was replaced with serum-free medium containing PtQDC. The scratched area was observed and photographed under an inverted microscope at 0 h, 12 h, and 24 h.

**Detection of oxidative stress-related indices:** To evaluate the inhibitory effect of PtQDC on cellular oxidative stress, the levels of reactive oxygen species (ROS) and lipid peroxides (LPOs) were detected. After 12Z cells were subjected to PtQDC treatment for 24 h, intracellular ROS levels were detected by using DCFH-DA. LPO levels were detected by fluorescence microscopy using a C11-BODIPY 581/591 probe (Beyotime S0043S). Moreover, glutathione peroxidase 4 (GPX4) activity, the ratio of reduced glutathione to oxidized glutathione (GSH/GSSG), and intracellular adenosine triphosphate (ATP) levels were detected by the abovementioned kits, which were operated in strict accordance with the instructions (Beyotime S0053, S0056, and S0027, respectively).

**Apoptosis analysis**: Apoptosis was detected by flow cytometry using Annexin V-FITC/PI double staining. After 12Z cells were subjected to PtQDC treatment for 24 h, the cells were collected by trypsin digestion, washed with PBS and resuspended in 1× binding buffer. Then, annexin V-FITC and PI staining solution were added and incubated for 15 min in the dark, followed by flow cytometry detection within 1 hour. The data were analyzed by FlowJo software to distinguish between live cells (annexin V-/PI-), early apoptotic cells (annexin V⁺/PI-), late apoptotic cells (annexin V ⁺/PI⁺) and necrotic cells (annexin V-/PI⁺), and the total apoptosis rate (early apoptosis rate + late apoptosis rate) was calculated.

**Experimental animals and EMs model construction**: The modeling method used was previously reported. Healthy 7-week-old female C57BL/6 mice were used for the experiments. After suitable rearing for one week, all the mice were injected subcutaneously with estrogen solution (150 μg/kg) twice for 4 days. The uteri were surgically removed from the donor mice, and the endometrial tissue was isolated and cut into small pieces (approximately 1 mm^3^). Recipient mice received intraperitoneal injections of these endometrial tissue blocks under anesthesia to establish an EMs model, whereas adipose tissue blocks were transplanted into the mice in the sham-operated group. Estrogen solution was injected subcutaneously into the back of the neck of each recipient mouse starting on the day of implantation and was repeated 4 days later for a total of two injections. The pharmacological interventions were initiated after the lesions were allowed to grow for one week.

**Experimental grouping and drug administration**: The successfully modeled mice were randomly divided into three groups: a model control group (Model group, given PBS), a low-dose PtQDC treatment group (PtQDC-L group), a high-dose PtQDC treatment group (PtQDC-H group); a sham-operated group (CON group) was also included. PtQDC was administered by intraperitoneal injection, and the intervention was performed for 2 consecutive weeks. At the end of the intervention, the peritoneal fluid was collected, the mice were sacrificed, and the ectopic lesion tissues in the abdominal cavity were dissected and collected for subsequent analysis. Some lesions were snap-frozen in liquid nitrogen for subsequent RNA-seq experiments, while other lesions were fixed with formalin for subsequent immunofluorescence staining.

**Evaluation of lesions and histological analysis**: The number of ectopic lesions in the abdominal cavity of each group of mice was recorded, and the longest (a) and shortest (b) diameters of the lesions were measured using Vernier calipers to calculate the lesion volume. The uterine adhesions caused by the lesions were also scored according to previously reported criteria.

**SOD and MDA assays**: After the mice were sacrificed, the tissue was precisely separated from the lesion, rinsed with saline, and mixed with precooled PBS in an ice bath to prepare a tissue homogenate. The tissue homogenate was centrifuged at 4°C, and the SOD activity and MDA content in the supernatant were determined. The SOD and MDA assay methods are described in the instruction manual (njjcbio A001-1; Servicebio G4302).

**Analysis of the levels of inflammatory and pain factors in the peritoneal fluid**: The peritoneal fluid of mice was collected by abdominal lavage. The levels of proinflammatory cytokines (IL-6, IL-1β, and TNF-α), an anti-inflammatory cytokine (IL-10), and kinin-releasing enzyme-related peptidase (KLK1) in the peritoneal fluids were detected by enzyme-linked immunosorbent assays (ELISAs), which were performed in strict accordance with the instructions of the ELISA kits (BOSTER EK0527, EK0394, EK0411, EK0417, and EK1586).

**Immunofluorescence staining**: After antigen repair of the paraffin sections was complete, primary antibodies (anti-TNF-α, anti-IL-1β, anti-IL-6, anti-IL-10, anti-VEGF, anti-CD31, anti-HIF-α, anti-CD10, anti-KLK1, anti-Substance P, and others) were added dropwise, and the slices were incubated at 4°C overnight. On the next day, the corresponding fluorescently labeled secondary antibody was added dropwise, the samples were incubated in the dark, the nuclei of the cells were stained with DAPI, and the samples were sealed. Images were acquired under a fluorescence microscope, and the fluorescence intensity of specific proteins was analyzed semiquantitatively using ImageJ software.

**RNA extraction, library construction, data preprocessing and differential expression analysis**: RNA-seq was performed by Shanghai Meiji Biomedical Technology Co. RNA extraction, library construction and sequencing, data processing and differential expression analysis are discussed in detail in the “Meiji RNA-seq Sequencing Project Technical Manual”. Raw data were subjected to quality control using FastQC and Trimmomatic to remove splice sequences and low-quality reads, after which HISAT2 was used to align high-quality clean data to a mouse reference genome. Gene expression was calculated using featureCounts and normalized by TPM. On the basis of the gene expression matrix, differential expression analysis was performed using the R package DESeq2 to screen for differentially expressed genes between the PtQDC treatment group and the model group. The screening criteria were a |log2(fold change)| ≥ 1 and a corrected P value (P adj) < 0.05.

**Functional enrichment analysis**: Gene Ontology (GO) enrichment analysis and Kyoto Encyclopedia of Genes and Genomes (KEGG) pathway enrichment analysis were performed on the identified differentially expressed genes. GO analysis included biological process (BP), cellular component (CC) and molecular function (MF) terms. Enrichment analysis was performed using the clusterProfiler R package, and a threshold of P value < 0.05 was used to filter significantly enriched entries or pathways.

**Protein–Protein Interaction (PPI) Network Construction and Core Gene Screening**: Differentially expressed genes were imported into the STRING database to identify protein–protein interactions, and a PPI network was constructed using Cytoscape software. By using the CytoHubba plug-in, we calculated the degree of each node in the network and selected the genes whose degree was higher than the average value as core candidate genes. Moreover, the MCODE plug-in was used to identify closely connected gene modules (clusters) in the network and calculate the MCODE score. The functions of core genes (e.g., Ptgs2, Postn, and Mmp3) in diseases were further analyzed in conjunction with the literature and database annotations.

**Validation of gene expression in a clinical datase**t: The GSE51981 dataset was downloaded from the GEO database (ncbi.nlm.nih.gov/geo/), which contains data for 26 patients with minimal/mild EMS and 48 patients with moderate/severe EMS. Differentially expressed genes were analyzed by the Limma package in R language. The expression of the core genes in this dataset was extracted and compared with that in normal tissues to verify the clinical relevance of the core genes. The Turku EMs Database (https://endometdb.utu.fi/), which contains 408 samples from a series of tissues, such as the endothelium, the peritoneum, and foci, was used, and the expression of PTGS2, POSTN, and MMP3 in clinical samples was analyzed.

Figure S1. UV-vis spectra of the PtQDC.

Figure S2. DLS results of PtQDC in the lesion tissue fluid after 0 days, 3 days and 7 days.

Figure S3. The body weight data recorded for mice in different treatment groups.

Figure S4. H&E staining of the heart, liver, spleen, lung and kidney of c57 mice in Model group and PtQDC_H group after 14 days of observation.

Figure S5. Statistical analysis of the relative fluorescence intensity of HIF-α.

Figure S6. Statistical analysis of the relative fluorescence intensity of CD10.

Figure S7. Maps of the significant KEGG pathways are shown (CYTOKINE-CYTOKINE RECEPTORINTERACTION and ECM-RECEPTOR INTERACTION).

Figure S8. Maps of the significant KEGG pathways are shown (CELL ADHESION MOLECULES and PI3K-AKTSIGNALING PATHWAY).

Figure S9. Maps of the significant KEGG pathways are shown (CHEMOKINESIGNALING PATHWAY and FOCAL ADHESION).
